# First report and genomic characterization of an *mcr-10.1*-carrying *Enterobacter kobei* strain isolated from a domestic kitchen sink in China

**DOI:** 10.1186/s12866-026-05205-2

**Published:** 2026-05-28

**Authors:** Xuejuan Liu, Ping Li, Yong Yan, Lei Gao, Miaomiao Jia, Shan Wang, Peiyan He, Guoying Zhu, Zhongwen Chen

**Affiliations:** Jiaxing Center for Disease Control and Prevention, Jiaxing, China

**Keywords:** *Enterobacter kobei*, *mcr-10.1*, Colistin resistance, Whole-genome sequencing, One Health

## Abstract

**Background:**

The Enterobacter cloacae complex (ECC), a member of the genus *Enterobacter* within the family Enterobacteriaceae, is an important opportunistic pathogen associated with nosocomial infections. Colistin is considered a last-resort antimicrobial for multidrug-resistant Gram-negative infections; however, the emergence and dissemination of plasmid-mediated colistin resistance genes, particularly *mcr* variants, have raised significant public health concerns. Among these, *mcr-10* has been increasingly detected in ECC isolates from diverse clinical and environmental sources, underscoring the importance of genomic surveillance to elucidate its genetic context and transmission potential.

**Methods:**

Swab samples were collected from a kitchen sink and cultured for bacterial isolation, followed by species identification using MALDI-TOF MS. Antimicrobial susceptibility was determined by the broth microdilution method, with results interpreted according to EUCAST and CLSI guidelines. Whole-genome sequencing was performed using a hybrid approach combining Illumina short-read and Oxford Nanopore long-read sequencing. Genome annotation and antimicrobial resistance gene detection were conducted using RAST and ResFinder, respectively. Multilocus sequence typing and plasmid replicon typing were performed using the MLST and PlasmidFinder tools. Comparative plasmid analysis was carried out with EasyFig and BLAST Ring Image Generator, while phylogenetic relationships were inferred using a core-genome-based phylogenetic analysis, and the resulting tree was visualized with iTOL.

**Results:**

*Enterobacter kobei* JX24083 was resistant to cefazolin, cefoxitin, and fosfomycin. Consistently, the isolate harbored the resistance genes *bla*_ACT-9_ and *fosA* on the chromosome. The colistin resistance gene *mcr-10.1* was identified on an IncFIB plasmid in the isolate; however, the isolate remained susceptible to colistin (MIC = 1 mg/L). Exposure to subinhibitory concentrations of colistin upregulated *mcr-10.1* expression in vitro. Comparative sequence analysis revealed a conserved *xerC–mcr-10* genetic context among *Enterobacter kobei* isolates harboring plasmid-borne *mcr-10.* Phylogenetic analysis of JX24083 together with 46 *mcr-10*-carrying ECC isolates from GenBank showed that several dominant sequence types, including ST681, ST125, and ST1, were distributed across different hosts and countries, indicating the potential for international dissemination.

**Conclusion:**

The first report of an *E. kobei* isolate from a domestic kitchen sink in China carrying a conserved *xerC–mcr-10* plasmid backbone, yet remaining susceptible to colistin, highlights the potential for silent dissemination of *mcr-10* within the Enterobacter cloacae complex (ECC) and underscores the need for continuous One Health-oriented surveillance of environmental reservoirs.

**Supplementary Information:**

The online version contains supplementary material available at 10.1186/s12866-026-05205-2.

## Background

The *Enterobacter cloacae* complex (ECC) is a group of opportunistic pathogens frequently associated with nosocomial infections. The genus *Enterobacter* is included among the ESKAPE pathogens (*Enterococcus faecium*,* Staphylococcus aureus*,* Klebsiella pneumoniae*,* Acinetobacter baumannii*,* Pseudomonas aeruginosa*,* and Enterobacter* species), a group of multidrug-resistant (MDR) organisms that pose a major threat to global public health [1, 2].The ECC has traditionally been described as a complex of *Enterobacter* species, including *E. cloacae*,* E. hormaechei*,* E. kobei*,* E. ludwigii*,* E. roggenkampii and E. asburiae.* However, its taxonomic composition has remained in flux. A recent whole-genome sequencing (WGS) study has redefined the ECC as a genetically diverse complex comprising multiple phylogenetic clades (A–V) [3]. The ECC is widely distributed in the environment and among in humans, animals, and plants, and can cause a variety of human infections, including sepsis, lower respiratory tract infections, urinary tract infections, and skin and soft tissue infections [4].

Owing to the increasing prevalence of extended-spectrum β-lactamase (ESBL)-resistant and carbapenem-resistant (CR) strains, ECC has become the third most common multidrug-resistant pathogen in the Enterobacteriaceae family causing nosocomial infections, following *Escherichia coli* and *Klebsiella pneumoniae* [5]. Due to the presence of its chromosomally encoded AmpC β-lactamase gene, ECC exhibits intrinsic resistance to ampicillin, amoxicillin, amoxicillin-clavulanic, and first- and second-generation cephalosporins [2]. Colistin is considered a last-resort antimicrobial for the treatment of multidrug-resistant Gram-negative bacterial infections. However, the increasing use of polymyxins has contributed to the emergence of resistance [6], and reports of colistin resistance in Enterobacteriaceae are steadily increasing [7, 8]. Colistin resistance is commonly mediated by mutations in chromosomal regulatory genes, including *pmrAB*, *phoPQ*, *crrAB*, *lpxACD*, and *mgrB*, which lead to lipid A modification of lipopolysaccharide (LPS) and consequently reducing colistin binding [9–11]. Following the first report of an *mcr-1*-harboring plasmid in China in 2016 [12], numerous *mcr* gene variants have been described. To date, ten *mcr* variants (*mcr-1* to *mcr-10*) are recognized, with a global distribution among bacterial species isolated from humans, animals, food, and environmental sources [13, 14].

*mcr-10*, located on an IncFIA-type plasmid, was first identified in a clinical isolate of *E. roggenkampii* from China. Introduction of this *mcr-10*-carrying plasmid into a colistin-susceptible strain resulted in a fourfold increase in the colistin MIC compared with that of the parental strain, indicating that *mcr-10* contributes to reduced susceptibility to colistin [15]. Since its initial identification, *mcr-10* has been detected in a range of reservoirs, including humans, hospital sewage, and food. Its widespread occurrence across clinical and environmental sources underscores the potential for transmission across human–environment interfaces and highlights the importance of monitoring environmental reservoirs for the dissemination of *mcr* [4]. In this study, we report the first isolation of an *mcr-10.1*-carrying *E. kobei* strain (JX24083) from a domestic kitchen sink in China. Whole-genome sequencing (WGS) was performed to characterize the genetic context of *mcr-10.1*, followed by comparative analyses with plasmid-borne *mcr-10*-carrying ECC strains retrieved from GenBank. In addition, phylogenetic analysis was conducted to assess the genetic relatedness of strain JX24083 to these publicly available *mcr-10*-positive ECC strains.

## Methods

### Bacterial isolation and identification

Swab samples were collected from the kitchen sink using sterile saline-moistened swabs, and inoculated into Gram-Negative Bacteria Broth (GN Broth) (Qingdao HopeBio Technology Co., Ltd., China) for enrichment. The enrichment cultures were subsequently streaked onto MacConkey (MAC) agar and Eosin Methylene Blue (EMB) agar (Qingdao HopeBio Technology Co., Ltd., China) and incubated at 36 °C for 24 h. Suspected colonies were identified by matrix-assisted laser desorption/ionization-time of flight mass spectrometry (MALDI-TOF MS) (Bruker, USA).

### Antimicrobial susceptibility testing

Antimicrobial susceptibility testing was performed to determine the susceptibility of the isolate to a panel of antimicrobial agents. Minimum inhibitory concentrations (MICs) were determined using the broth microdilution method in cation-adjusted Mueller–Hinton broth (CAMHB). A commercial antimicrobial susceptibility testing panel (Sensitire, Thermo Scientific, UK) was used to determine susceptibility to the following antimicrobial agents: amikacin, ampicillin, cefazolin, cefepime, cefotaxime, cefoxitin, ceftazidime, cefuroxime, chloramphenicol, ciprofloxacin, colistin, ertapenem, gentamicin, imipenem, meropenem, tetracycline, amoxicillin–clavulanate, ampicillin–sulbactam, and polymyxin B. MICs were read using the VIZION Susceptibility Test Reader (Trek Diagnostic Systems Ltd., United Kingdom). For fosfomycin, agar dilution was performed using Mueller–Hinton agar supplemented with 25 mg/L glucose-6-phosphate (Macklin Biochemical Co., Ltd., China) and twofold serial dilutions of fosfomycin (0.8 − 256 mg/L) (MedChemExpress, USA). The isolate using a 0.5 McFarland suspension in triplicate and incubated at 37 °C for 16–20 h prior to interpretation. Fosfomycin-free control plates were included. Interpretation of breakpoints for colistin and fosfomycin followed European Committee on Antimicrobial Susceptibility Testing guidelines(EUCAST) ( v15.0), while those for the other antimicrobial agents were interpreted using the Clinical and Laboratory Standards Institute guidelines (CLSI )(M100-2025). Escherichia coli ATCC 25922 was used as the quality control. All experiments were performed with three biological replicates.

### Colistin induction and quantitative real-time reverse transcription PCR (qRT–PCR) assay

For the induction assay, strain JX24083 was initially inoculated into Luria–Bertani (LB) broth and grown at 37 °C with shaking at 180 rpm until the optical density at 600 nm (OD₆₀₀) reached approximately 0.5. The culture was then diluted 1:100 into fresh LB broth supplemented with colistin at final concentrations of 0.25 mg/L (1/4 MIC) and 0.5 mg/L (1/2 MIC). LB medium without colistin was used as the control. The culture was incubated at 37 °C for 8 h, and harvested by centrifugation at 12,000 × g for 2 min at 4 °C to collect bacterial pellets. Total RNA was extracted using a total RNA extraction kit (QIAGEN GmbH, Germany) according to the manufacturer′s instructions, and RNA concentration was measured using a NanoDrop 2000 spectrophotometer (Thermo Fisher Scientific, USA). cDNA was synthesized using the PrimeScript™ RT Reagent Kit (TaKaRa Bio Inc., Japan). qRT–PCR was performed using TB Green^®^ Premix Ex Taq™ II (TaKaRa Bio Inc., Japan) according to the manufacturer′s protocol. The *16 S* rRNA gene was used as an internal control for normalization. The relative expression level of *mcr-10* was calculated using the 2^⁻ΔΔCt^ method [16]. Detailed primer sequences are provided in Supplementary Table S1.

### Whole-genome sequencing and bioinformatics analysis

Genomic DNA was extracted following the manufacturer′s instructions using the QIAamp DNA Mini Kit (Qiagen, Germany). DNA concentrations were quantified using the Qubit™ 1× dsDNA HS Assay Kit (Invitrogen, USA), followed by magnetic bead-based DNA purification (Hangzhou Matridx Biotechnology Co., Ltd., China). For short-read sequencing, the library was prepared using a library preparation kit (Hangzhou Matridx Biotechnology Co., Ltd., China) and quantified with the KAPA Library Quantification Kit (Roche, Switzerland). The library was sequenced on the Illumina NextSeq 550 platform using a Mid Output v2 Reagent kit (300 cycles) (Illumina, Inc., USA). For long-read sequencing, the library was prepared using the Sequencing Ligation Kit and loaded onto an R10.4.1 flow cell for sequencing on the Nanopore MinION platform (Oxford Nanopore Technologies, Oxford, UK). Raw Nanopore reads were quality filtered using NanoFilt (v.2.8.0) [17]. Hybrid assembly of raw Illumina short reads and trimmed MinION long reads was performed using Flye (v2.9), and the contigs obtained were polished using Pilon (v.1.24) [18]. Species identification was performed using JSpeciesWS [19]. Genome annotation of *E. kobei* was conducted using RAST [20]. Antibiotic resistance genes were identified using ResFinder from the Center for Genomic Epidemiology [21]. Multilocus sequence typing (MLST) of the ECC was performed using the MLST online tool [22], and plasmids replicon types were determined using PlasmidFinder [23]. Comparative plasmid analyses were performed using EasyFig (v.2.2.5) [24], and BLAST Ring Image Generator (v.0.95) [25]. The online tool oriTfinder was used to identify the origin of transfer (oriT) and associated conjugative elements [26]. Genome sequences carrying *mcr-10* retrieved from GenBank were re-annotated using Prokka (v1.14.6) [27], and the core genome alignment was generated using Roary (v3.12.0) [28]. An approximately maximum-likelihood phylogenetic tree was constructed using FastTree (v2.0.0) [29]. The resulting Newick-format tree was visualized using iTOL (v7) [30].

## Results

### Identification of an *mcr-10.1*-carrying *E. kobei* strain

Samples were collected from a kitchen sink in Jiaxing, China. The isolate was initially identified as *Enterobacter* spp. using an MALDI-TOF MS. Subsequent genetic analysis confirmed it as *E. kobei*, and it was designated as strain JX24083. The strain had a MIC value of 1 mg/L for colistin, classifying it as colistin-sensitive according to EUCAST guidelines. It was resistant to ampicillin, cefazolin, cefoxitin, fosfomycin, amoxicillin/clavulanate, and ampicillin/sulbactam (Table 1).

**Table 1:** Antimicrobial susceptibility testing of JX24083 isolate in this study

Antimicrobial agents	MIC(mg/L)	Antimicrobial susceptibility/(S/I/*R*)
Amikacin	≤ 4	S
Ampicillin	=32	R
Cefazolin	>32	R
Cefepime	≤ 1	S
Cefotaxime	≤ 0.12	S
Cefoxitin	>64	R
Ceftazidime	≤ 0.5	S
Cefuroxime	=8	S
Chloramphenicol	=8	S
Ciprofloxacin	≤ 0.01	S
Colistin	=1	S
Ertapenem	≤ 0.25	S
Gentamicin	≤ 1	S
Imipenem	=0.5	S
Meropenem	≤ 0.12	S
Tetracycline	= 4	S
Amoxicillin/Clavulanate	=64	R
Ampicillin Sodium/Sulbactam	=16	R
Polymyxin B	≤ 0.25	S
Fosfomycin	= 16	R

*S* susceptible, *I* intermediate, *R* resistant

### Genetic structure and characterization of *E. kobei* strain JX24083

**Fig. 1:**
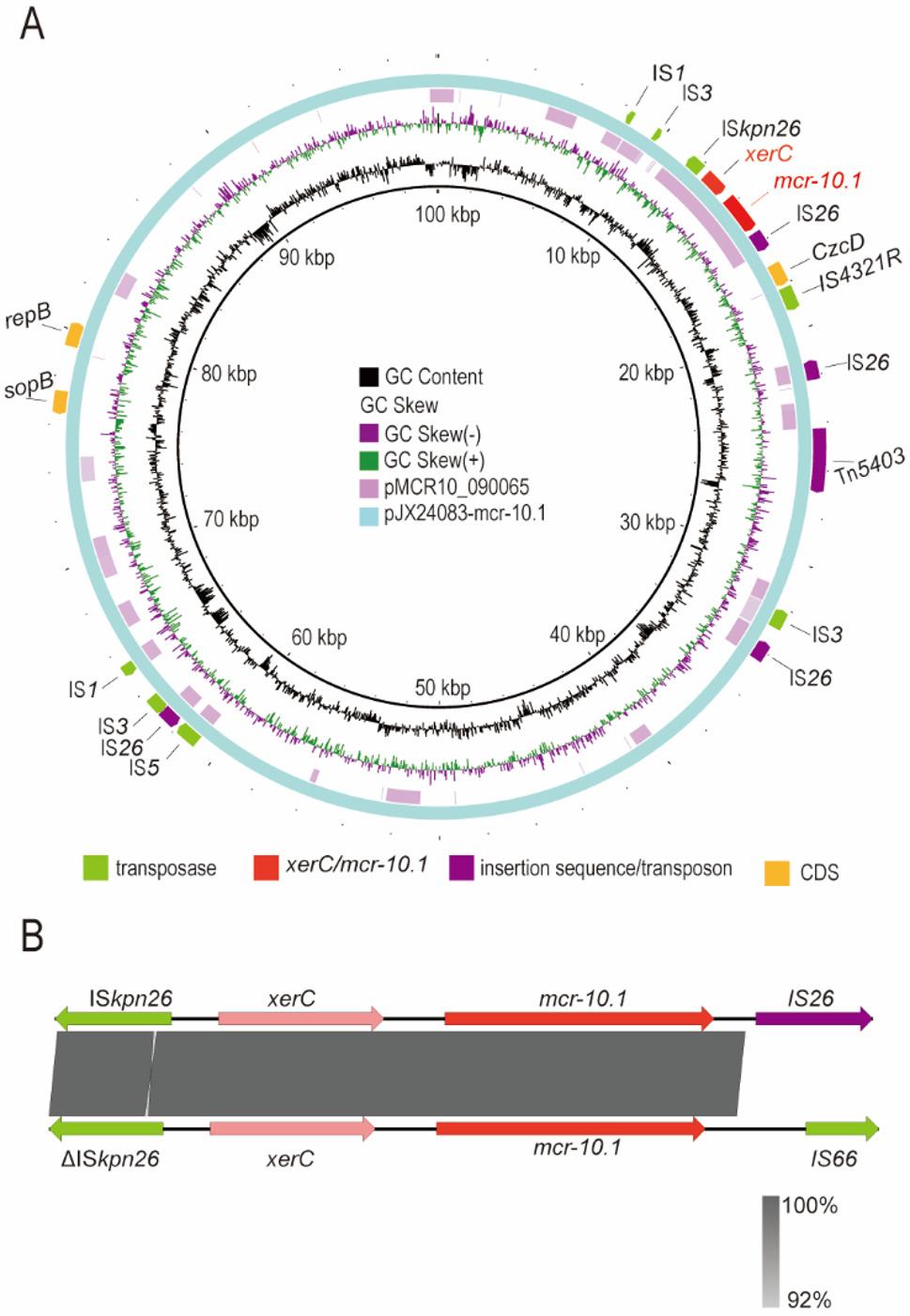
Comparative genomic analysis of plasmids pJX24083-mcr-10.1 and pMCR10_090065. **A** Schematic map of plasmids pJX24083-mcr-10.1 and pMCR10_090065. GC content and GC skew are displayed from the inner to the outer circles, with genes shown as arrows indicating the transcriptional direction of the ORFs. Sequence similarity is indicated by colored regions. **B** Comparative analysis of the genetic environments of mcr-10.1 in plasmids pJX24083-mcr-10.1 and pMCR10_090065. xerC is shown in pink, mcr-10.1 in red, transposases in green and insertion sequences in purple.

Complete genomes of isolate JX24083 was obtained using short-read and long-read whole-genome sequencing. Hybrid assembly of strain JX24083 using Nanopore long reads and Illumina short reads generated 2.38 Gb of clean data, with an average coverage depth of approximately 464×. The *de novo* hybrid assembly yielded a complete genome consisting of a circular chromosome (5,022,238 bp) and a circular plasmid (100,086 bp), with a contig N50 of 5,022,238 bp. The average nucleotide identity (ANI) between strain JX24083 and the *E. kobei* reference strain (DSM 13645) from GenBank was 98.71%. The *E. kobei* strain was assigned to ST520 by MLST. The strain harbored a *mcr-10.1*-carrying plasmid, designated pJX24083-mcr-10.1, which was located on contig 2. This plasmid had a total length of 100,086 bp, was of the IncFIB(K) replicon type, and carried resistance gene *mcr-10.1* (Table 2). Plasmid annotation revealed that the region upstream of *mcr-10.1* contained *xerC*, while the downstream region harbored the IS*26* (Fig. 1A).

**Table 2:** Resistance genes of JX24083 and 7 *mcr-10*-carrying isolates from GenBank

Strain	Species	Sequence type	Source	Plasmid type	Resistance genes
JX24083	*E. Kobei*	520	Kitchen sink	**IncFIB(k)** ^a^	*bla*_ACT−9,_ *fosA*, ***mcr-10.1***
STW0522-51	*E. Kobei*	770	Sewage water	**repE**, IncFIB(PECLA), IncN3, IncX10	*bla*_ACT−9,_ *fosA*, ***mcr-10.1***, *aadA11*,* bla*_OXA−2_, *bla*_CTX−M−62_
SCLZS19	*E. Kobei*	122	NA	IncHI2, **IncFⅡ**, **IncFIB(k)**, IncHI2A, IncP1, IncP6, IncQ2	*fosA*,* bla*_ACT−9_, ***mcr-10***, *mcr-9.2*,* ant (2’’)-la*,* bla*_KPC−2_, *sul*,* aadA2b*,* bla*_CTX−M−9_, *bla*_TEM−40_, *bla*_TEM−150_, *bla*_TEM−10_, *bla*_TEM_*-*_1 A_, *qnrS2*
11778-yvys	*E. Kobei*	280	Human	**IncFIB(pECLA)**	*bla*_*ACT−9*_, ***mcr-10.2***, *fosA*
Ek72	*E. Kobei*	125	Human	**IncFIB(pECLA)**, **IncFⅡ(pECLA)**, IncX3	*bla*_ACT−9_, *bla*_NDM,_ *bla*_SHV−12_, ***mcr-10.1***, *fosA*
EXB6	*E. Kobei*	125	NA	**IncFIB(k)**, **IncFⅡ(pECLA)**	*bla*_ACT−9,_ ***mcr-10.1***, *fosA*
Mu208	*E. Kobei*	125	Human	**IncFIB(k)**, **IncFⅡ(pECLA)**	*bla*_ACT−9,_ ***mcr-10.1***, *fosA*
EXB31	*E. Kobei*	125	NA	**IncFIB(k)**, **IncFⅡ(pECLA)**, repA	*bla*_ACT−9_ ***mcr-10.1***, *fosA*

^a^The replicon types and resistance genes of *mcr-10*-carrying plasmids are shown in bold

### Comparison of the genetic characteristics of *mcr-10*-carrying plasmids in ECC

**Fig. 2:**
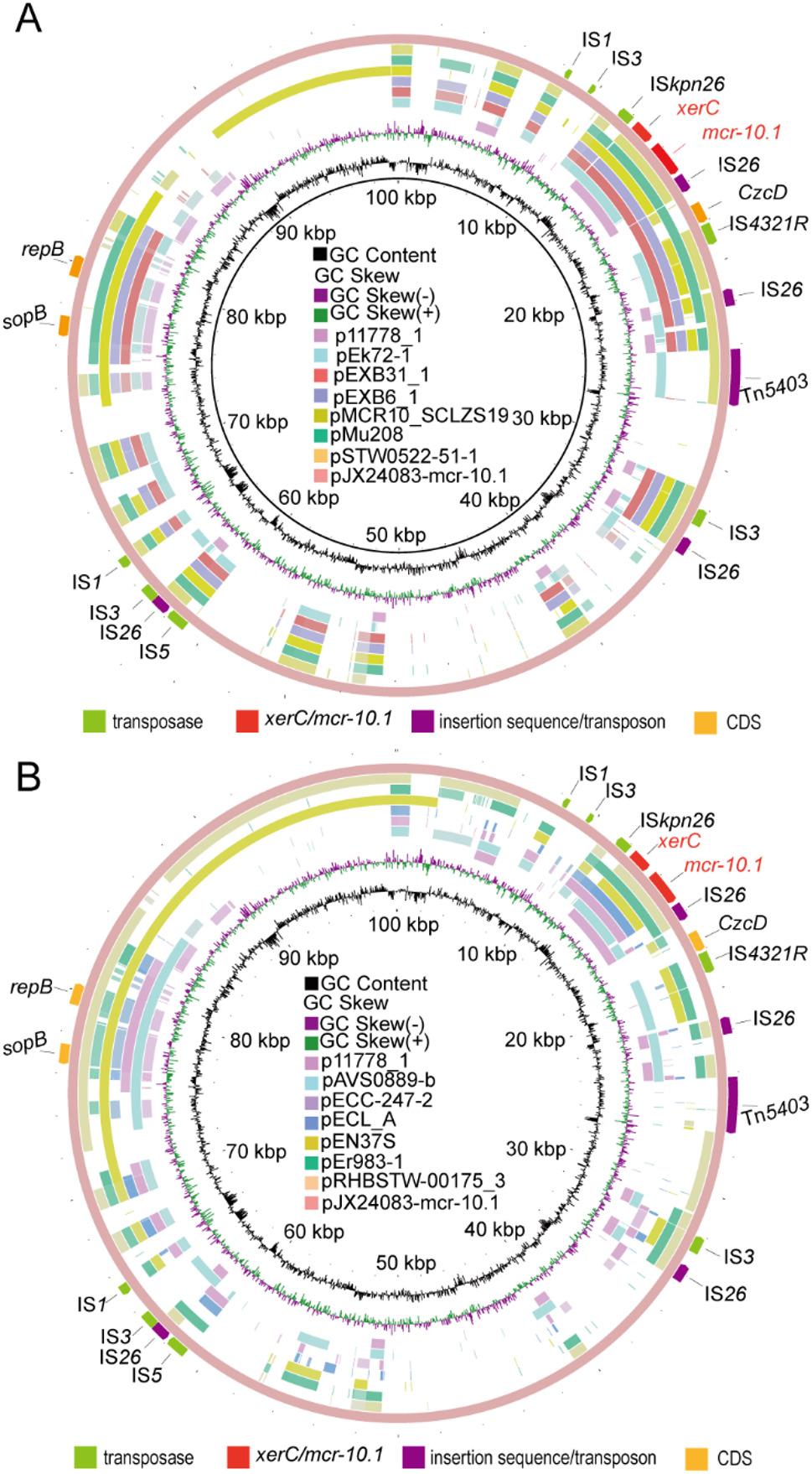
Schematic maps of mcr-10-harboring plasmids retrieved from GenBank. **A** Comparative genomic analysis of mcr-10-carrying plasmids in E. kobei strains. **B** Comparative genomic analysis of mcr-10-carrying IncFIB plasmids in ECC strains. GC content and GC skew are displayed from the inner to the outer circles, and genes are shown as arrows indicating the transcriptional direction of the coding sequences. Sequence similarity is indicated by colored regions

**Fig. 3:**
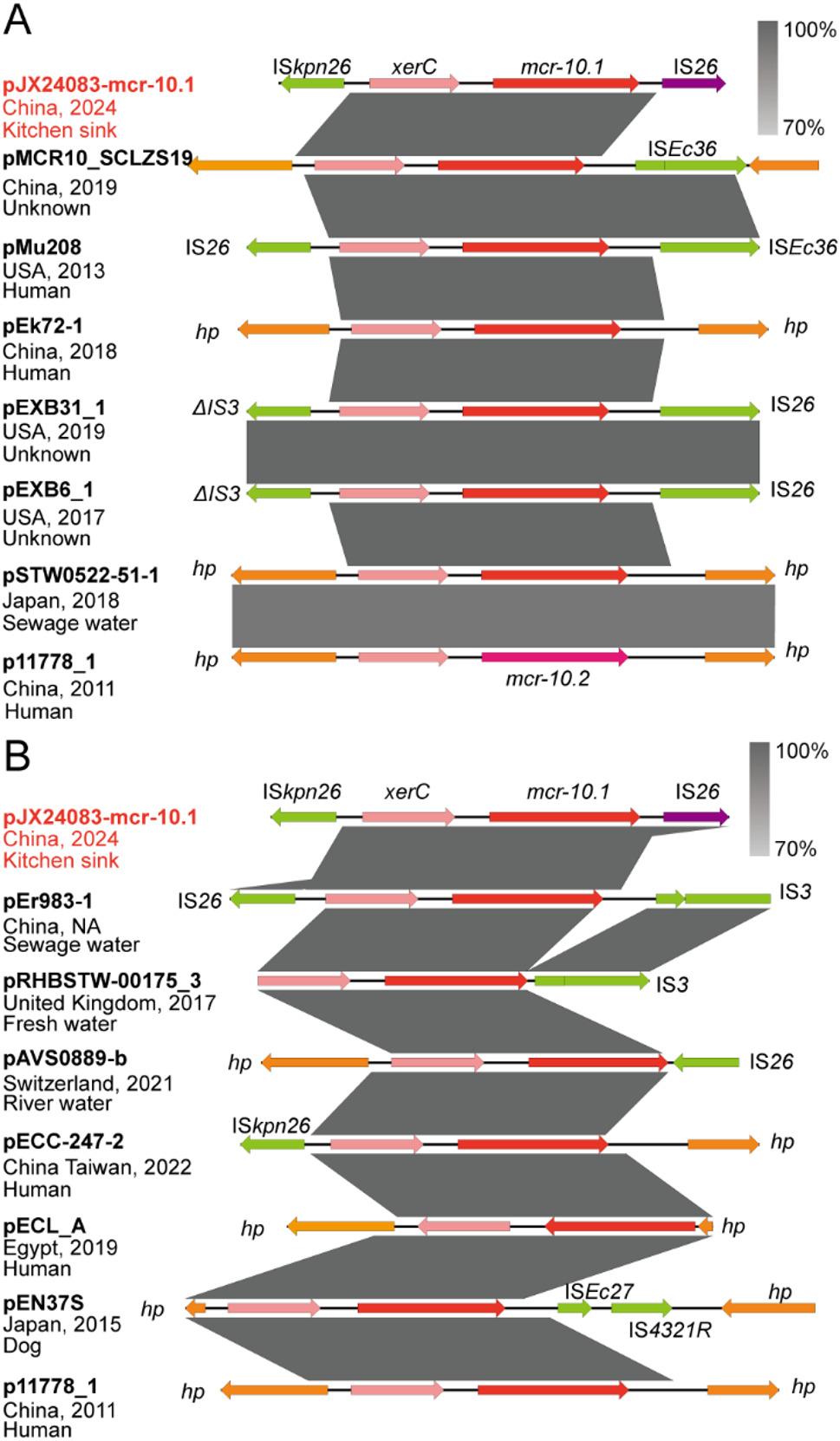
Schematic representation and comparison of the genetic environments of mcr-10-positive plasmids. **A** Comparative analysis of the genetic environments of mcr-10.1 in plasmids from E. kobei strains retrieved from GenBank. **B** Comparative analysis of the genetic environments of mcr-10.1 in IncFIB plasmids from ECC strains retrieved from GenBank. Genes are color-coded as follows: xerC, pink; mcr-10.1, red; mcr-10.2, light red; insertion sequences, purple; transposase, magenta; green; hypothetical proteins, orange

A BLASTn comparison between pJX24083-mcr-10.1 and pMCR10_090065 showed 99.85% nucleotide identity with 26% query coverage. The *mcr-10.1* in pMCR10_090065 is flanked by a truncated IS*Kpn26* upstream of *xerC* and an IS*66* family transposase downstream, as pMCR10_090065 was the first plasmid identified to carry *mcr-10.1* (Fig. 1B). Seven other *E. kobei* strains carrying *mcr-10* located on plasmids were retrieved from GenBank: four ST125 *E. kobei* strains carrying plasmids pEK72-1, pEXB6_1, pMu208 and pEXB31_1, a ST770 strain carrying pSTW0522-51-1, a ST122 strain carrying pMCR10_SCLZS19, and a ST280 strain carrying p11778. A *xerC–mcr-10* structure was found in all these plasmids (Fig. 2A). Similar to pJX24083-mcr-10.1, the upstream and downstream regions of the *xerC–mcr-10* structure in pMU208 were flanked by IS*Ec36* and IS*26*, respectively, whereas those in pEXB31_1 and pEXB6_1 were flanked by ΔIS*3* and IS*26*. However, the upstream region of *mcr-10* in pMCR10 contained a hypothetical protein, while both the upstream and downstream regions of *mcr-10* in pSTW0522-51-1, p11778, and pEk72-1 harbored hypothetical proteins (Fig. 3A). Sequence alignment analysis of the IncFIB-type plasmids carrying *mcr-10* (p11778, pAVS0889-b, pECC-247-2, pECL, pEN37S, pEr983-1, pRHBSTW-00175) revealed a conserved *xerC–mcr-10* structure (Fig. 3B). Additionally, the upstream and downstream regions of *mcr-10* in these plasmids were flanked by different transposases or hypothetical proteins (Fig. 3B). However, pAVS0889-b carries a disrupted *mcr-10* with a 29-bp insertion at position 64; IS*26* is at the 5’ end, and *xerC* is downstream of *mcr-10*. In contrast, the *mcr-10* in plasmid pRHBSTW-00175-3 has a 94-bp deletion at the 3’ end and is downstream of IS*Ec36*.

### Upregulation of *mcr-10.1* under subinhibitory colistin exposure and plasmid transferability prediction

To understand *E. kobei* strain JX24083 is susceptible to colistin despite harboring the *mcr-10* gene, a colistin induction assay was performed. Compared with the antibiotic-free control, the expression of *mcr-10* was upregulated by 2.6-fold and 3.2-fold upon exposure to subinhibitory concentrations of colistin at 1/4 MIC and 1/2 MIC, respectively (Fig. S1). These results indicate that *mcr-10* carried by plasmid pJX24083-mcr-10.1 is inducible under colistin selective pressure. Similar phenomena have been reported in colistin-susceptible *Escherichia coli* strains harboring *mcr-10.1*, where the MIC significantly increases upon exposure to colistin [31]. 

To assess the transferability of plasmid pJX24083-mcr-10.1, its sequence was analyzed using oriTfinder. No conjugative elements, including oriT, relaxase, T4CP, or T4SS, were identified, suggesting that the plasmid is non-conjugative [26].

### Phylogenetic analysis of *mcr-10*-carrying ECC isolates

**Fig. 4:**
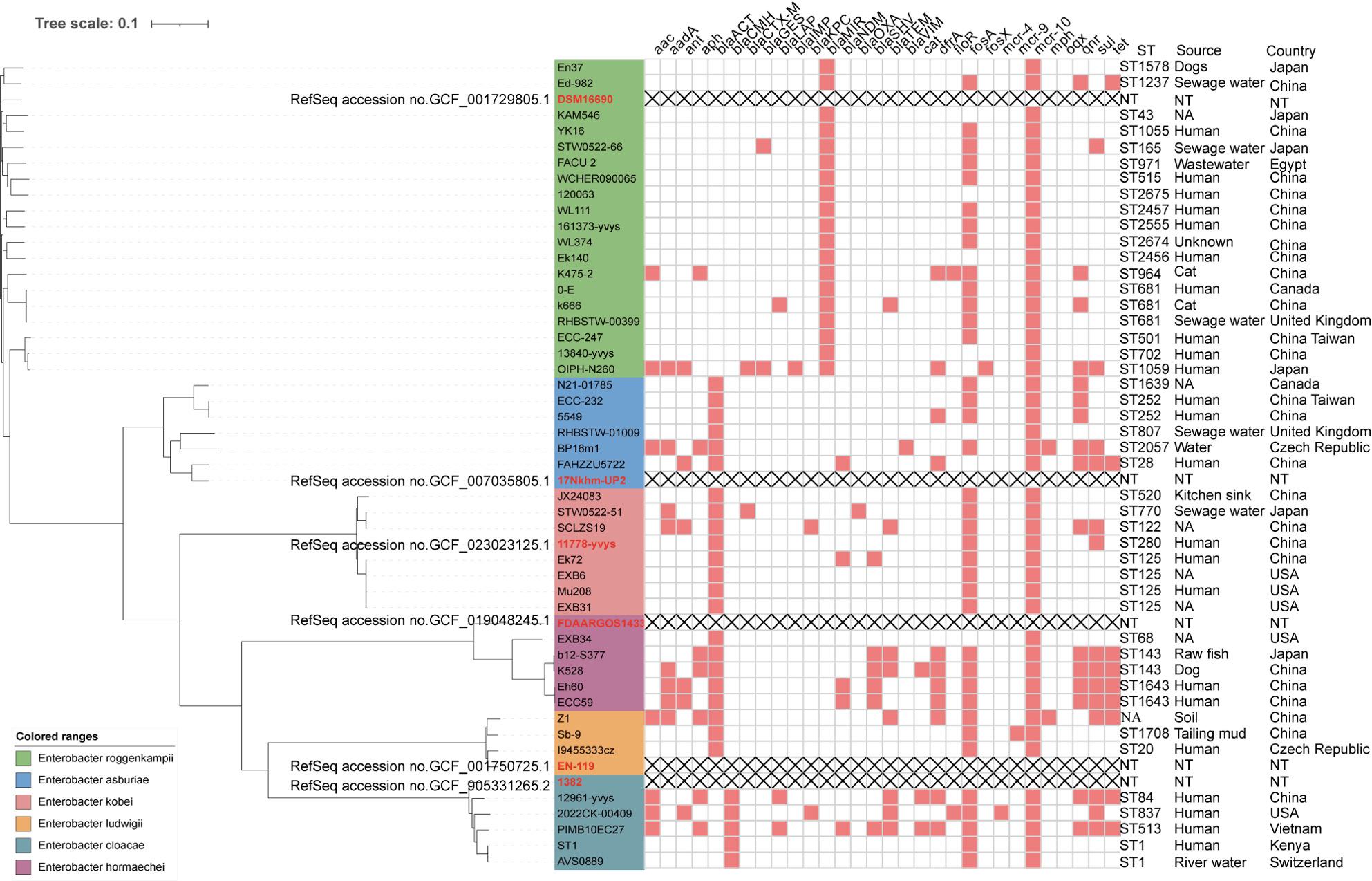
Core-genome-based phylogenetic tree of 46 ECC isolates. Different species within the ECC are indicated by different colors. The heatmap depicts the distribution of antimicrobial resistance genes among the isolates, with red squares indicating gene presence. Corresponding STs, isolation sources, and countries of origin are indicated on the right. In the figure, information on reference genome strains, resistance genes, and MLST types was not analyzed

Plasmids carrying *mcr-10* were retrieved from GenBank, with a total of 67 plasmids identified. These plasmids were derived from 67 strains representing 13 species, including *E. cloacae, E. roggenkampii, E. asburiae, E. hormaechei, E. ludwigii, E. kobei, Enterobacter sp., Escherichia coli, Citrobacter freundii, Citrobacter portucalensis, Klebsiella pneumoniae, Klebsiella quasipneumoniae, and Raoultella ornithinolytica* (Table S2). Full-length genome sequences of *mcr-10*-positive ECC strains were retrieved from GenBank, and a phylogenetic tree based on core-genome-based phylogenetic analysis was constructed. In the phylogenetic tree (Fig. 4) the 46 ECC strains belonging to different species were clustered into 7 major clades. These clades included *E. roggenkampii* (*n*=19), *E. kobei* (*n*=8), *E. asburiae* (*n*=6), *E. cloacae* (*n*=5), *E. hormaechei* (*n*=5), and *E. ludwigii* (*n*=3). These strains were isolated from China (*n*=25), Japan (*n*=6), Egypt (*n*=1), the United Kingdom (*n*=2), the Czech Republic (*n*=2), Canada (*n*=2), the USA (*n*=5), Vietnam (*n*=1), Kenya (*n*=1), and Switzerland (*n*=1). The majority of isolates (37/46) carried the fosA gene, which confers resistance to fosfomycin. However, *bla*_CMH_ was carried exclusively by *E. cloacae*, whereas *bla*_MIR_ was detected only in *E.* *roggenkampii*. Additionally, *E. asburiae*,* E. hormaechei*,* E. kobei*, *and E. ludwigii* all harbored *bla*_ACT_. Three ST681 strains, which clustered together phylogenetically, were isolated from Canada, China, and the United Kingdom. Similarly, ST125, ST143, and ST1 each formed distinct phylogenetic clusters and were identified in multiple countries. Two ST1 strains—one isolated from a human in Kenya and the other from river water in Switzerland—carried the same resistance genes (including *mcr-10*), suggesting potential transmission of resistant ECC between humans and the environment.

## Discussion

ECC is a multidrug-resistant nosocomial pathogen that imposes a significant health burden on humans. Although colistin is regarded as the last line of defense for antibiotic therapy, the misuse of antibiotics has led to the widespread dissemination of *mcr*-carrying plasmids among humans, animals, and the environment worldwide [15, 32, 33]. In this study, an *E. kobei* strain carrying *mcr-10.1* was first isolated from a domestic kitchen sink in China, and its resistance phenotype was characterized. Whole-genome sequencing (WGS) was performed, followed by analysis of the genetic composition of the resistance plasmid. A comparative analysis of *mcr-10*-carrying plasmids in GenBank was conducted to clarify the potential transmission routes of antimicrobial resistance plasmids between humans and the environment.

The JX24083 isolate harbored *fosA*, *bla*_ACT−9_, and *mcr-10.1*, with *fosA* and *bla*_ACT−9_ located on the chromosome and *mcr-10.1* on an IncFIB (K) plasmid. IncF plasmids, a common type of plasmid found in Enterobacteriaceae, are primarily associated with β-lactam resistance genes. *bla*_ACT_ is a predominant β-lactamase gene in ECC isolates from Southwest China. Notably, *bla*_ACT−9_, a chromosomally encoded AmpC β-lactamase gene, exhibited species-specific distribution and was exclusively detected in *E. kobei* [2, 34]. *fosA* is a metalloenzyme that confers fosfomycin resistance by catalyzing glutathione conjugation to the drug, leading to its inactivation [35]. *mcr-10* is widely disseminated through various Inc plasmid types, which are generally conjugative and mobile, highlighting their potential spread across different niches in the One Health spectrum [36, 37]. The pJX24083-mcr-10.1 plasmid harbors a *xerC–mcr-10.1* structure, with IS*Kpn26* upstream of *xerC* and IS*26* downstream of *mcr-10.1* (Fig. 1A). XerC belongs to the CRE recombinase family of tyrosine recombinases and is involved in site-specific recombination. Notably, the *xerC–mcr-10* structure is highly conserved among *mcr-10*-carrying Enterobacteriaceae, suggesting that the dissemination of *mcr-10* may be linked to this *XerC*-mediated recombination system [15, 38–42]. Comparison of the genetic makeup of *mcr-10*-carrying IncFIB plasmids from ECC strains retrieved from GenBank revealed that the downstream region of *mcr-10* in six plasmids contained insertion sequences or transposases (two with IS*26*, two with IS*Ec36*, and one with ΔIS*Ec36*), while the remaining three plasmids contained a hypothetical protein downstream of *mcr-10* (Fig. 2B). Among the *E. kobei* strains carrying *mcr-10* retrieved from GenBank, two plasmids had *mcr-10* flanked by IS*Ec36* downstream, while the other three contained a hypothetical protein downstream (Fig. 3A). Consistent with our findings, a study by Yi et al. analyzed 145 *xerC–mcr-10* structures in GenBank, 45 of which contained IS*Ec36* and IS*26*, the primary mobile elements mediating *mcr-10* dissemination [4].

Although the JX24083 strain harbored *mcr-10.1*, it remained susceptible to colistin (MIC = 1 mg/L). Notably, *mcr-10.1* has been identified in colistin-susceptible isolates of *Escherichia coli*, ECC, and *Klebsiella pneumoniae* [31, 43–45]. Colistin acts as a cationic antimicrobial agent, targeting lipopolysaccharide (LPS) in the bacterial outer membrane to exert antibacterial effects [46]. Gram-negative bacilli can develop resistance through chromosomal mutations that mediate the addition of 4-amino-4-deoxy-L-arabinose (L-Ara4N) and/or phosphoethanolamine (PEtN) to the phosphate groups of lipid A, thereby reducing the ability of colistin to bind to lipopolysaccharide (LPS) [47, 48]. The response regulators PhoP and PmrA of the two-component systems PhoP–PhoQ and PmrA–PmrB are activated upon phosphorylation, leading to the regulation of *arnBCADTEF* and *pmrCAB* expression. These genes mediate LPS modification with L-Ara4N and PEtN [49]. Although *mcr-10* has been shown to be associated with colistin resistance, the identification of *mcr-10*-carrying strains susceptible to colistin suggests that *mcr-10* may fail to confer resistance due to limited expression. Additionally, some *mcr* variants do not confer resistance to colistin, which may be due to low plasmid copy numbers or reduced gene expression [39]. In our study, the expression of mcr-*10.1* was upregulated upon colistin exposure, indicating that the plasmid-borne *mcr-10.1* gene may be inducible by colistin. Overall, existing studies have not fully clarified the mechanism underlying the susceptibility of *mcr-10*-carrying isolates to colistin. However, the possibility of interspecies transmission due to *mcr* variants has been discussed, and the transmission of *mcr-1*0-carrying isolates remains a major concern [45].

Phylogenetic analysis of the genomes of *mcr-10*-carrying isolates retrieved from GenBank revealed that *E. roggenkampii* strains that share the common sequence type (ST681) clustered in the same clade, with three isolates originating from a human in Canada, a cat in China, and sewage water in England, respectively. Similarly, four *E. kobei* isolates of ST125 (from China and the United States), two *E. kobei* isolates of ST143 (from animals in China and Japan), two *E. hormaechei* isolates of ST1643 (from humans in China), and two *E. cloacae* isolates of ST1 (from humans and river water in Kenya and Switzerland) were identified. These findings indicate that *mcr-10*-carrying isolates may be transmitted among humans, animals, and the environment within the One Health spectrum.

Also, this study has several limitations. Notably, the sample size was limited, and only a single *mcr-10*-carrying isolate from the ECC was characterized. Therefore, the prevalence and dissemination patterns of *mcr-10* within the ECC could not be comprehensively assessed. Future studies involving larger collections of environmental and clinical isolates are warranted to better understand the prevalence and transmission dynamics of *mcr-10* in this species complex.

## Conclusion

To our knowledge, this is the first report of an *E. kobei* strain carrying *mcr-10.1* isolated from a domestic kitchen sink in Jiaxing, China. The strain exhibited resistance to β-lactam antibiotics but remained susceptible to colistin, indicating that further studies are needed to clarify the role of *mcr-10* in colistin resistance and its underlying mechanisms. Comparative genomic analysis of *mcr-10*-carrying plasmids revealed a conserved *xerC–mcr-10* structure, whereas the flanking regions showed considerable variability due to different insertion sequences and transposases. The dissemination of ECC strains harboring *mcr-10* and additional antimicrobial resistance genes across human, environmental, and animal-associated settings underscores their potential public health risk. From a One Health perspective, continued surveillance of the spread of *mcr-10*-carrying plasmids in environmental reservoirs is warranted.

## Supplementary Information

Supplementary Material 1.

Supplementary Material 2.

## Data Availability

The dataset generated in this study are publicly available in the NCBI database under BioProject accession number PRJNA1393866. The complete plasmid sequence of pJX24083-mcr-10.1 has been deposited in GenBank under accession number PX766137.
