# Supplementary material for: First report and genomic characterization of an mcr-10.1-carrying Enterobacter kobei strain isolated from a domestic kitchen sink in China
Source: BMC Microbiol. 2026 May 28;26:673. doi: 10.1186/s12866-026-05205-2 (PMC13403616; doi:10.1186/s12866-026-05205-2)
Supplement: Supplementary file 1 — Supplementary Material 1. [file 12866_2026_5205_MOESM1_ESM.docx]

**Supplementary data**

**Table. S1** The RT-qPCR primer sequences used in this study.

| **Primers** | **Sequences (5′→3′) *** | **Amplicon size (bp)** | **Annealing temperature (°C)** |
| --- | --- | --- | --- |
| *16s* | F: TCCACGATTACTAGCGATTC  R: AGCAAGCGGACCTCATAA | 87 | 55 |
| *mcr-10* | F: ACCTGCTATGACGATGTTAT  R: TGAAGTGACGATGCTCTG | 124 | 55 |

F, forward; R, reverse; bp, base pair

**Reference**

Zhang S, Sun H, Lao G, Zhou Z, Liu Z, Cai J, Sun Q: Identification of Mobile Colistin Resistance Gene mcr-10 in Disinfectant and Antibiotic Resistant Escherichia coli from Disinfected Tableware. *Antibiotics (Basel, Switzerland)* 2022, **11**(7).

**
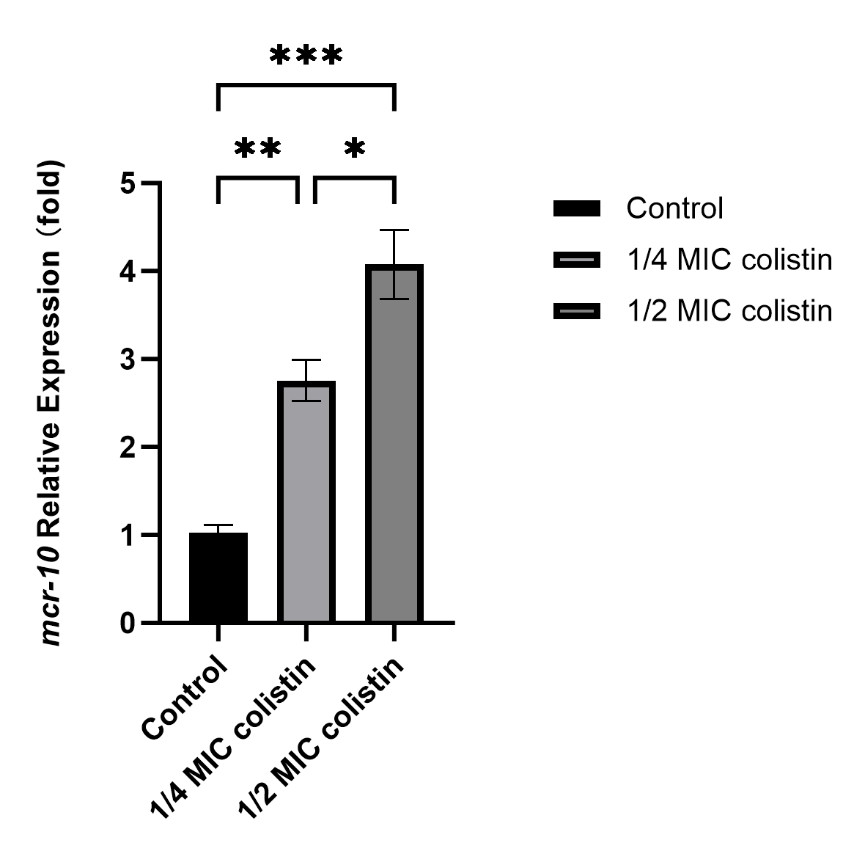
**

**Fig. S1 Relative expression of *mcr-10* in *E. kobei* JX24083 following colistin exposure.** Data are presented as mean ± SEM from three independent biological replicates. Data were analyzed using one-way ANOVA followed by Tukey’s post-hoc multiple comparisons test. **P < 0.01, ***P < 0.001 compared with the untreated control group; *P < 0.05 compared with the 1/4 MIC colistin-treated group.
